# Vascular Endothelial Growth Factor A Contributes to Increased Mammalian Respiratory Epithelial Permeability Induced by Pasteurella multocida Infection

**DOI:** 10.1128/spectrum.04554-22

**Published:** 2023-03-14

**Authors:** Lin Lin, Jie Yang, Dajun Zhang, Qingjie Lv, Fei Wang, Peng Liu, Mixue Wang, Congcong Shi, Xi Huang, Wan Liang, Chen Tan, Xiangru Wang, Huanchun Chen, Brenda A. Wilson, Bin Wu, Zhong Peng

**Affiliations:** a State Key Laboratory of Agricultural Microbiology, The Cooperative Innovation Center for Sustainable Pig Production, College of Veterinary Medicine, Huazhong Agricultural University, Wuhan, China; b Hubei Hongshan Laboratory, Wuhan, China; c Department of Microbiology, University of Illinois at Urbana-Champaign, Urbana, Illinois, USA; Emory University School of Medicine

**Keywords:** *Pasteurella multocida*, vascular endothelial growth factor A, VEGFA, hypoxia-inducible factor 1α, HIF-1α, mammalian respiratory epithelial permeability, pathogenesis

## Abstract

Pasteurella multocida infection can cause significant zoonotic respiratory problems in both humans and animals, but little is known about the mechanisms used by P. multocida to invade and cross the mammalian respiratory barrier. In this study, we investigated the influence of P. multocida infection on the dysfunction of the respiratory epithelial barrier. *In vivo* tests in mouse infection models demonstrated that P. multocida infection significantly increased epithelial permeability and increased the expression of vascular endothelial growth factor A (VEGFA) and endothelial nitric oxide synthase (eNOS) in murine tracheae and lungs. In murine lung epithelial cell (MLE-12) models, P. multocida infection decreased the expression of tight junctions (ZO-1) and adherens junctions (β-catenin and E-cadherin) proteins but induced the activation of hypoxia-inducible factor 1α (HIF-1α) and VEGFA signaling. When the expression of HIF-1α is suppressed, the induction of VEGFA and ZO-1 expression by P. multocida infection is decreased. We also found that intervention of HIF-1α and VEGFA signaling affected infection outcomes caused by respiratory bacteria in mouse models. Most importantly, we demonstrate that P. multocida infection increases the permeability of human respiratory epithelial cells and that this process is associated with the activation of HIF-1α and VEGFA signaling and likely contributes to the pathogenesis of P. multocida infection in humans.

**IMPORTANCE** The mammalian respiratory epithelium forms the first line of defense against infections with P. multocida, an important zoonotic respiratory pathogen. In this study, we found that P. multocida infection increased respiratory epithelial permeability and promoted the induction of the HIF-1α–VEGFA axis in both mouse and murine cell models. Similar findings were also demonstrated in human respiratory epithelial cells. The results from this study provide important knowledge about the pathogenesis of P. multocida causing infections in both animals and humans.

## INTRODUCTION

Respiratory infections pose serious health and socioeconomic problems. It has been estimated that respiratory infections are responsible for the deaths of 3.96 million people worldwide annually (1). In agriculture, respiratory disorders such as avian influenza (AI), bovine respiratory disease (BRD), and porcine respiratory disease complex (PRDC) have resulted in hundreds of millions of economic losses in the global livestock industry (2–4). The mammalian respiratory epithelium forms the first line of defense against invasion and damage caused by respiratory pathogens (5, 6). Respiratory epithelial cells are connected to their neighbors by cell-to-cell junctions, including tight junctions (TJs), adherens junctions (AJs), gap junctions, and desmosomes, and these interconnections are the central components of the host respiratory epithelial barrier (6). To invade the body and cause disease, it is necessary for respiratory pathogens to induce permeability in host respiratory epithelial barriers to promote disease pathogenesis (7). However, the detailed mechanisms used by respiratory pathogens to invade host respiratory epithelial barriers remain unclear.

Vascular endothelial growth factor A (VEGFA) is an endothelium-specific growth factor that accelerates angiogenesis and vascular permeability (8, 9), and endothelial nitric oxide (NO) synthase (eNOS) plays an essential role in mediating angiogenesis and endothelial function induced by VEGFA, through the production of NO (10). The production of VEGFA is regulated by hypoxia-inducible factor 1 (HIF-1), a major transcription factor composed of two subunits (α and β) that induces the cellular response to hypoxic conditions (8, 11). It has been found that HIF-1–VEGFA signaling is involved in the development of many diseases, including chronic airway inflammatory diseases (11), Clostridium difficile-induced enteritis (12), and pneumococcal meningitis (13). However, it remains to be further explored whether HIF-1–VEGFA signaling contributes to the modulation of epithelial permeability induced by respiratory bacterial infection.

Pasteurella multocida is an important zoonotic respiratory pathogen that is capable of causing respiratory diseases in multiple animals as well as in humans (14). Isolates belonging to this Gram-negative bacterial species are commonly divided into five capsular serogroups/genotypes (A, B, D, E, and F) (15–17). Generally, P. multocida capsular serogroups/genotypes A, D, and F are frequently associated with respiratory syndromes in both animals and humans (17). As a well-known respiratory pathogen, P. multocida most commonly causes infections via respiratory tracts (18). However, this bacterium can cause damage to multiple organs in addition to the respiratory organs, including the hearts, spleens, livers, kidneys, guts, and even brains, of both humans and animals after infections via respiratory tracts (19–21). In addition, it is common in clinical cases to recover P. multocida from organs in addition to respiratory organs (22–24). Therefore, we hypothesize that P. multocida invades and crosses the host respiratory epithelial barrier during infection. However, to our knowledge, related studies of the pathogenesis of P. multocida infection have not been reported. In this study, we investigated the influence of P. multocida infection on the dysfunction of the respiratory epithelial barrier in epithelial cell models and mouse models. Our results demonstrated that P. multocida isolates of both human and animal origins could increase the permeability of respiratory epithelial barriers during infection, and this process was associated with the activation of the HIF-1α–VEGFA pathway.

## RESULTS

### Pasteurella multocida infection increases the permeability of the respiratory epithelial barrier and promotes the production of VEGFA in mouse infection models.

**FIG 1:**
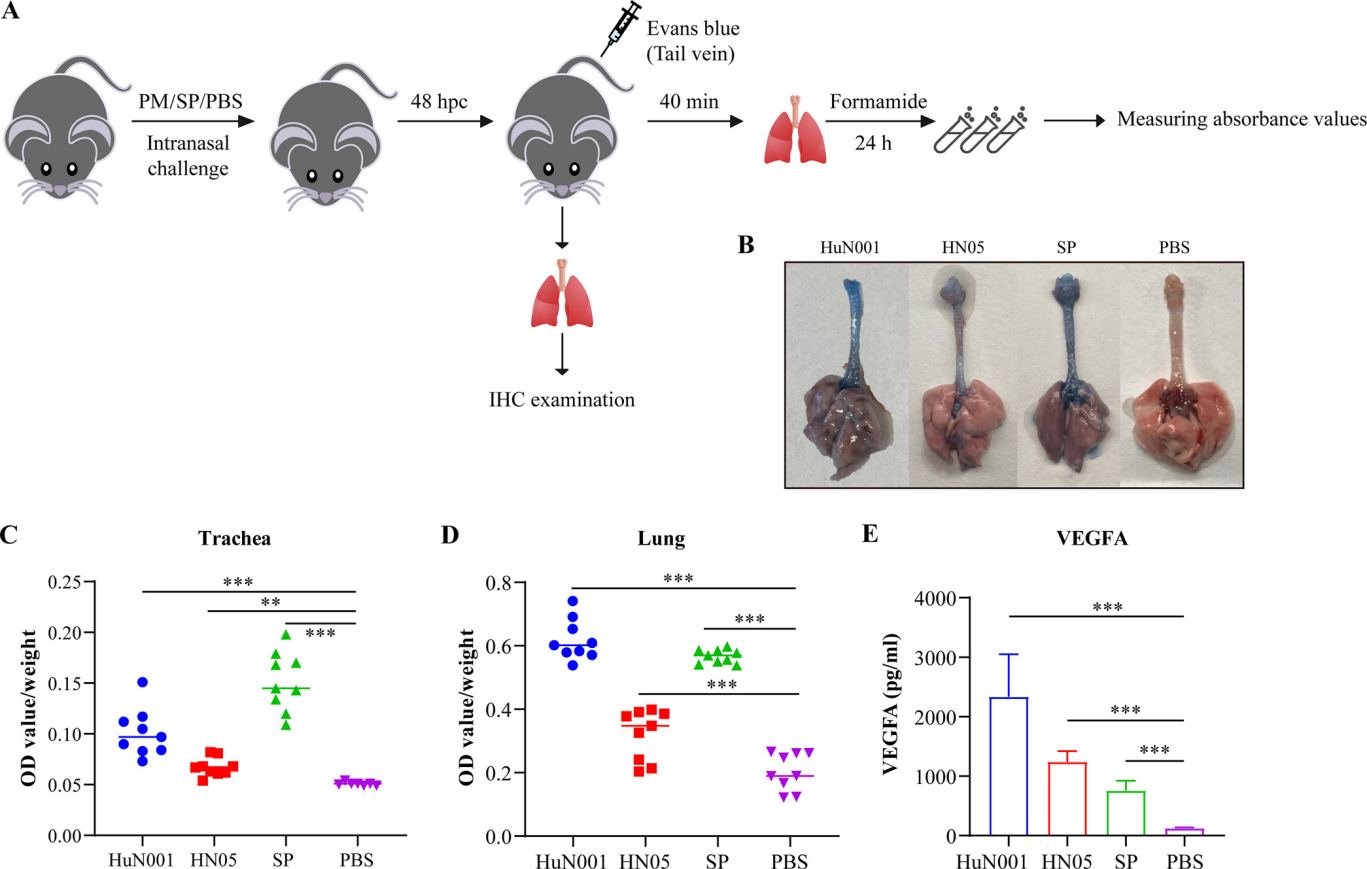
Pasteurella multocida infection increases respiratory vascular permeability in mice. (A) Study design of the mouse experiment. Mice were inoculated intranasally with P. multocida (PM) HuN001 or HN05, S. pneumoniae (SP) D39, or PBS, and infection by S. pneumoniae was included as a quality control for the assay. After 48 h, each of the mice was administered Evans blue through the tail vein to assess the vascular permeability of the tracheae and lungs. After 40 min, the mice were euthanized, and dyes in the tracheae and lungs were extracted using formamide. Changes in vascular permeability were evaluated by measuring the absorbance values (OD_620_) of the solutions. In parallel, the tracheae and lungs of bacterium-infected mice without tail vein injection of Evans blue were also collected for immunohistochemical (IHC) examinations. (B) Visualization of the tracheae and lungs obtained from mice inoculated with P. multocida strains (HuN001 and HN05), S. pneumoniae, or the control (PBS), showing respiratory vasculature and staining with Evans blue dye. (C) Quantification of Evans blue in the tracheae obtained from bacterium-infected mice and control mice. (D) Quantification of Evans blue in the lungs obtained from bacterium-infected mice and control mice. (E) Expression of vascular endothelial growth factor A (VEGFA) in the tracheae and lungs obtained from bacterium-infected mice and control mice. hpc, hours postchallenge.

**FIG 2:**
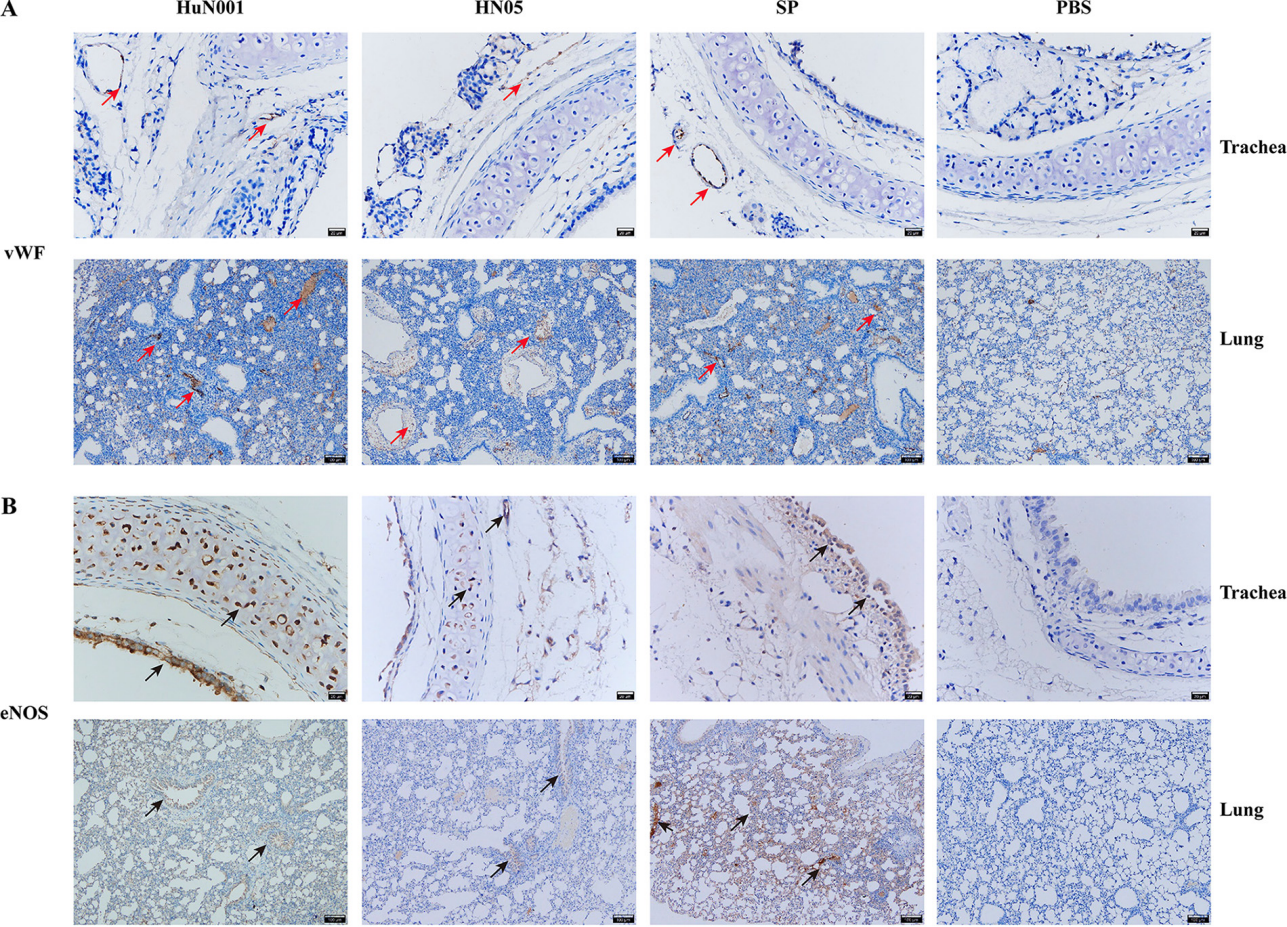
Pasteurella multocida infection increases vWF and eNOS expression in mouse tracheae and lungs. Shown are images from immunohistochemical examinations of the tracheae and lungs obtained from bacterium-infected mice, compared to control mice, treated as described in the legend of Fig. 1. (A) Expression of von Willebrand factor (vWF) (shown with the red arrows) in the tracheae (top) and lungs (bottom) obtained from bacterium-infected mice (HuN001, HN05, or S. pneumoniae) and control mice (PBS). (B) Expression of endothelial nitric oxide synthase (eNOS) (shown with the black arrows) (marked using the eNOS polyclonal antibody) in the tracheae (top) and lungs (bottom) obtained from bacterium-infected mice and control mice.

To explore whether respiratory bacterial infection influences the permeability of mammalian respiratory epithelial barriers, C57BL/6 mice (~5 to 6 weeks old) were intranasally inoculated with P. multocida strains from different hosts (HuN001 from a human and HN05 from a pig), Streptococcus pneumoniae strain D39, or phosphate-buffered saline (PBS) (Fig. 1A). At 48 h postinoculation, we assessed vascular permeability the in tracheae and lungs of infected mice by the systemic injection of Evans blue, which is an azo dye that has a very high affinity for serum albumin and has been widely used to assess the changes in the permeability of physiological barriers in different disease models (12). These results revealed that the vascular permeability of the tracheae and lungs was remarkably increased in mice infected with respiratory bacteria compared to the control mice treated with PBS (*P* < 0.001 for both tracheae and lungs) (Fig. 1B, C, and D). The production of VEGFA in the tracheae and lungs of experimental mice, assayed using enzyme-linked immunosorbent assays (ELISAs), revealed increased VEGFA levels in bacterium-infected mice (Fig. 1E). Immunohistochemical (IHC) analysis using von Willebrand factor (vWF) antibody revealed the increased expression of vWF in the tracheae and lungs of the infected mice compared to PBS-treated mice (Fig. 2A); IHC staining with eNOS polyclonal antibody also showed the increased production of eNOS in the tracheae and lungs of the bacterium-infected mice compared the PBS-treated mice (Fig. 2B). These findings indicated that increased vascular permeability occurred in the tracheae and lungs during P. multocida infection.

### P. multocida infection induces disruption of barrier functions of murine respiratory epithelial cells by promoting HIF-1α–VEGFA signaling.

**FIG 3:**
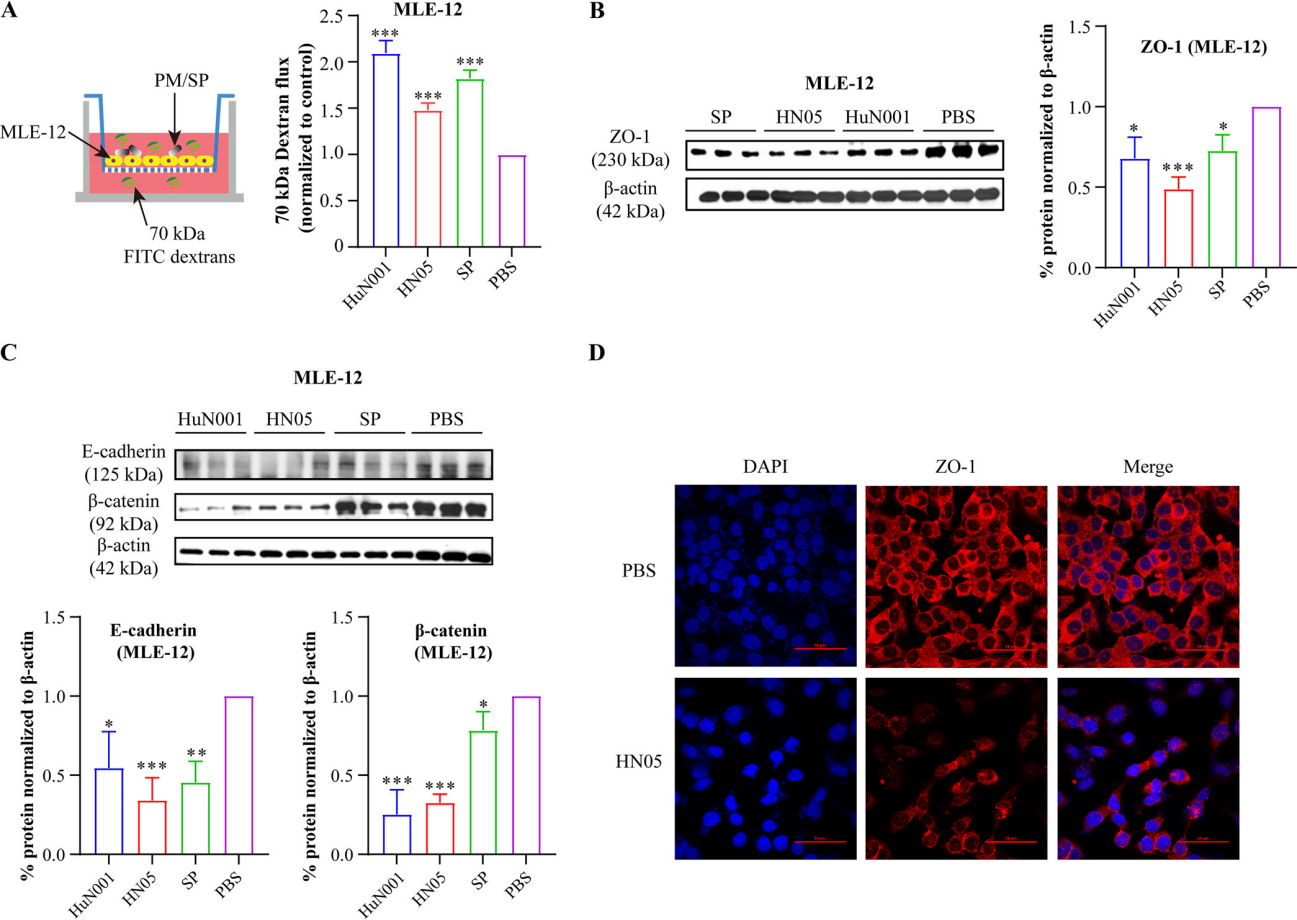
Pasteurella multocida infection affects the vascular permeability of murine lung epithelial cells (MLE-12). (A) Dextran-based transwell permeability assay showing the increase in the vascular permeability of MLE-12 cells induced by P. multocida infection. Infection by S. pneumoniae was included as a quality control for the assay. (B) Expression of ZO-1 in bacterium-infected MLE-12 cells compared to PBS-treated cells. At the right is a plot showing quantifications of the Western blot from cell extracts in triplicate. (C) Western blots demonstrating the expression of β-catenin and E-cadherin in bacterium-infected cells and PBS-treated cells. (D) Confocal laser scanning microscopy images showing the reduced expression of ZO-1 in MLE-12 cells infected with P. multocida HN05 compared to control PBS-treated cells. DAPI, 4′,6-diamidino-2-phenylindole.

We next investigated the impact of respiratory bacterial infection on the permeability of mammalian respiratory epithelial barriers. To achieve this, we first performed a dextran-based transwell permeability assay in murine lung epithelial cells (MLE-12) using fluorescently labeled dextrans of 70 kDa to obtain size-specific permeability (Fig. 3A). The results showed that infection with P. multocida HuN001 or HN05 induced a significant increase in the permeability of MLE-12 cells (Fig. 3A). We next detected the expression of junction molecules (ZO-1, β-catenin, and E-cadherin), and the results showed that the expression of these proteins was significantly decreased in bacterium-infected cells compared to PBS-treated cells (Fig. 3B and C). Visualization by using confocal laser scanning microscopy also revealed that P. multocida infection decreased the expression of the key TJ protein ZO-1 (Fig. 3D).

**FIG 4:**
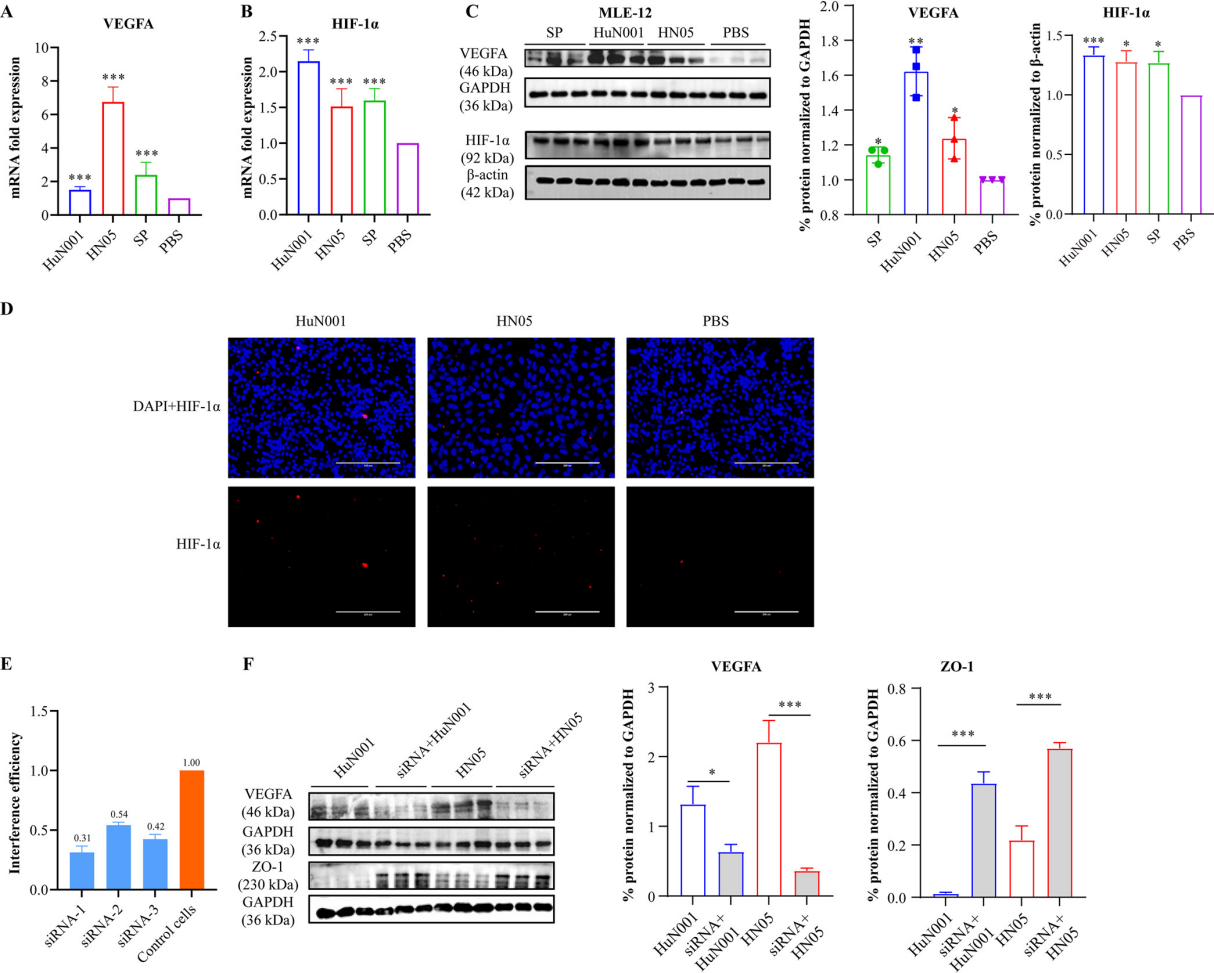
Expression of VEGFA and HIF-1α in murine lung epithelial cells (MLE-12) infected with Pasteurella multocida for 6 h. (A) Transcription of VEGFA in bacterium-infected cells compared to PBS-treated cells, as determined by qPCR. (B) Transcription of HIF-1α in bacterium-infected cells compared to PBS-treated cells, as determined by qPCR. (C, left) Western blots demonstrating the expression of VEGFA and HIF-1α in bacterium-infected cells compared to PBS-treated cells. (Right) Plot showing quantifications of the Western blot from cell extracts in triplicate. (D) Increased transcription of HIF-1α in bacterium-infected cells compared to PBS-treated cells, as determined by immunofluorescence. (E) Efficacy of three specific siRNAs in suppressing the expression of HIF-1α in MLE-12 cells. (F) Expression of VEGFA and ZO-1 in HIF-1α-suppressed cells after infection with P. multocida strains (HuN001 and HN05) for 6 h. At the right are plots showing quantifications of the Western blot (left) from cell extracts in triplicate (control in duplicate). Infection by S. pneumoniae was included as a quality control for the assay.

To explore the involvement of the HIF-1α–VEGF signaling pathway in mammalian respiratory barrier dysfunction during respiratory bacterial infection, we examined the expression levels of VEGFA and HIF-1α in MLE-12 cells after bacterial infection for 6 h. The results showed that the expression of HIF-1α and VEGFA increased in respiratory bacterium-infected murine respiratory epithelial cells compared to those treated with PBS (Fig. 4A to D). Next, we knocked down HIF-1α expression in MLE-12 cells and determined the expression levels of VEGFA in the cells after P. multocida infection. The results revealed that the expression of VEGFA decreased in HIF-1α knockdown cells compared to that in wild-type cells after bacterial infection (Fig. 4E and F). In contrast, the expression of the TJ protein ZO-1 in HIF-1α knockdown cells did not decrease as much after infection (Fig. 4F). The above-described findings indicated that P. multocida infection led to the disruption of the barrier functions of murine respiratory epithelial cells by downregulating the expression of TJ and AJ proteins between the cells, and this process was associated with the activation of the HIF-1α–VEGF pathway induced by bacterial infection.

### Treatment with a VEGFA-blocking antibody attenuates vascular permeability and offers protection against P. multocida in mouse infection models.

**FIG 5:**
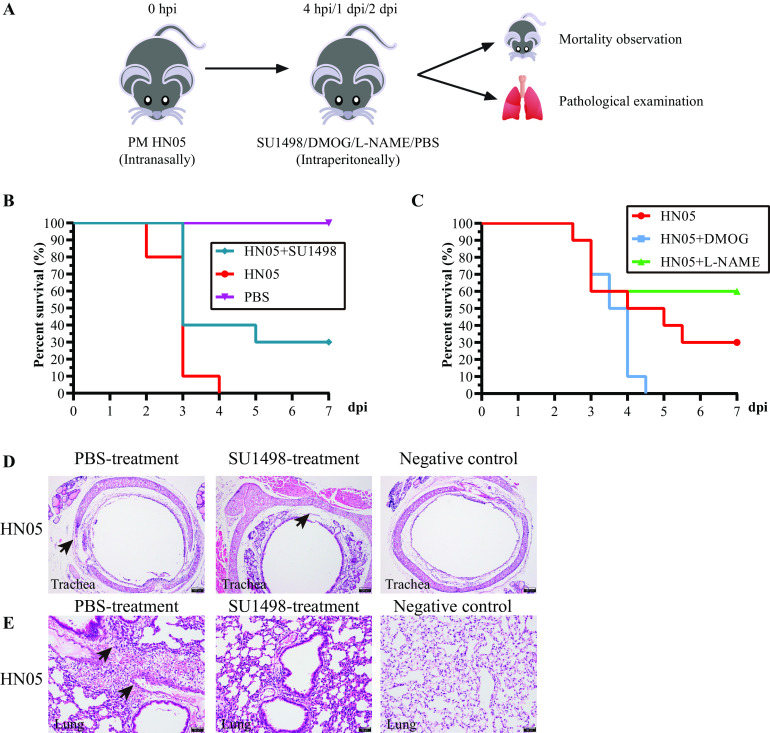
Effect of intervention of HIF-1α–VEGFA signaling on infections caused by respiratory bacteria in mouse models. (A) Study design of the mouse experiments. (B) Survival curves for P. multocida-infected mice with or without treatment with the VEGFR-2 inhibitor (SU1498). (C) Survival curves of P. multocida-infected mice with or without treatment with the HIF-1α agonist (DMOG) or the eNOS inhibitor (L-NAME). (D) Histological analysis of the tracheae obtained from P. multocida-infected mice with or without treatment with the VEGFR-2 inhibitor (SU1498) (HE) (magnification, ×100). (E) Histological analysis of the lungs obtained from P. multocida-infected mice with or without treatment with the VEGFR-2 inhibitor (SU1498) (HE) (magnification, ×100). Histological damage is indicated with black arrows.

We next explored whether blocking the HIF-1α–VEGFA signaling pathway could relieve respiratory bacterial infections. To achieve this, C57BL/6 mice (~5 to 6 weeks old) were first intranasally infected with P. multocida HN05, and at 4 h postinfection (hpi), 1 day postinfection (dpi), and 2 dpi, each of the infected mice received treatment with the vascular endothelial growth factor receptor 2 (VEGFR-2) inhibitor SU1498, the HIF-1α agonist DMOG, the eNOS inhibitor L-NAME, or PBS by intraperitoneal injection (Fig. 5A). Mortality results revealed that treatment with SU1498 or L-NAME decreased the rate of death of mice induced by P. multocida infection, while treatment with DMOG increased the mortality rate (Fig. 5B and C). We also compared the pathological damage caused by HN05 in the tracheae and lungs of mice treated with SU1498 to that in mice without treatment. Histological examinations showed that P. multocida infection induced severe drops in epithelial cell and inflammatory cell infiltration into the murine tracheae, and treatment with SU1498 reduced this damage (Fig. 5D). In murine lungs, P. multocida infection caused severe thickening of alveolar walls, fibrosis, as well as the exudation of inflammatory cells and red cells in alveolar spaces (Fig. 5E). In contrast, treatment with SU1498 reduced this damage (Fig. 5E). The above-described findings indicated that the HIF-1α–VEGFA signaling pathway contributed to the pathogenesis of P. multocida causing respiratory infections.

### Infections with P. multocida isolates from multiple hosts alter barrier functions and activate the HIF-1α–VEGFA pathway in human respiratory epithelial cells.

**FIG 6:**
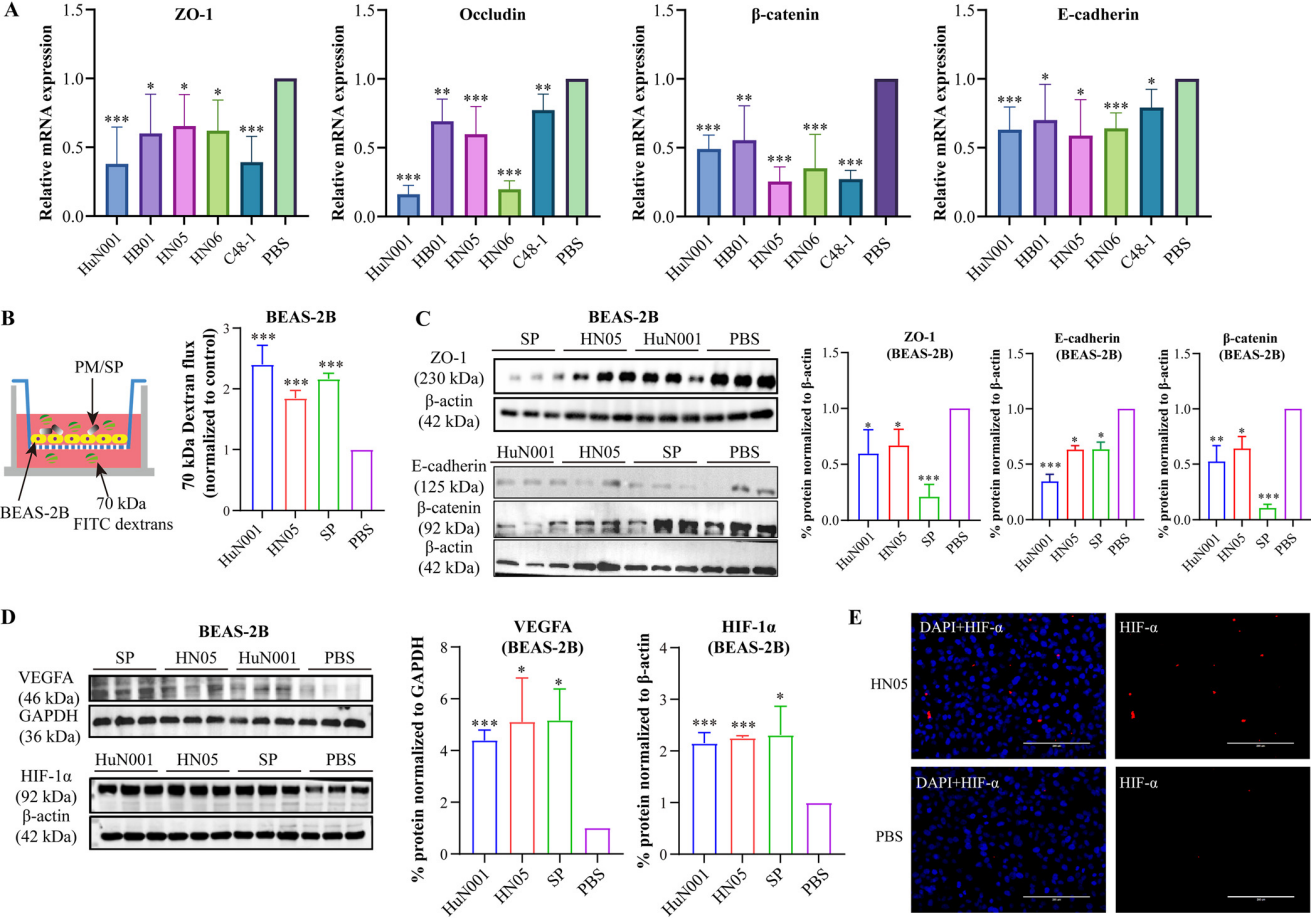
Induction of vascular permeability and VEGFA–HIF-1α signaling in human respiratory epithelial cells (BEAS-2B) infected with Pasteurella multocida strains. (A) Transcription of tight junction proteins (ZO-1 and occludin) and adherens junction proteins (β-catenin and E-cadherin) in cells infected with P. multocida isolates from different hosts (HuN001 from a human, HB01 from cattle, HN05 and HN06 from pigs, and C48-1 from poultry) or PBS-treated cells, as evidenced by qPCR analysis. (B) Dextran-based transwell permeability assay showing the increase in the vascular permeability of BEAS-2B cells induced by P. multocida infection. Infection by S. pneumoniae was included as a quality control for the assay. (C) Expression of ZO-1, β-catenin, and E-cadherin in bacterium-infected cells compared to PBS-treated cells. At the right are plots showing quantifications of the Western blot (left) from cell extracts in triplicate. (D) Expression of VEGFA and HIF-1α in bacterium-infected cells compared to PBS-treated cells. At the right are plots showing quantifications of the Western blot (left) from cell extracts in triplicate. (E) Immunofluorescence analysis of the transcription of HIF-1α in bacterium-infected cells and PBS-treated cells. DAPI, 4′,6-diamidino-2-phenylindole.

We used human respiratory epithelial cells (BEAS-2B) as a model to tentatively explore the mechanisms of P. multocida pathogenesis. Since our recent work demonstrated that P. multocida isolates from different hosts are capable of adhering to and invading BEAS-2B cells (25), we first examined the expression levels of TJ proteins (ZO-1 and occludin) and AJ proteins (β-catenin and E-cadherin) in BEAS-2B cells infected with P. multocida isolates from different hosts (HuN001 from a human, C48-1 from poultry, HB01 from cattle, or HN05 and HN06 from pigs). The results revealed that P. multocida from different hosts showed the capacity to invade human respiratory epithelial cells (Fig. 6A). Dextran-based transwell permeability assays using fluorescently labeled dextrans of 70 kDa showed a significant increase in the permeability of BEAS-2B cells to dextran upon infection with the HuN001 or HN05 strain (Fig. 6B), which was also supported by the decreased expression of junction molecules (ZO-1, β-catenin, and E-cadherin) (Fig. 6C). Following this, we examined the activation of HIF-1α–VEGFA signaling and found that the expression levels of VEGFA and HIF-1α were increased in cells infected with P. multocida compared to PBS-treated cells (Fig. 6D and E).

## DISCUSSION

In this study, we explored the influence of P. multocida infection on the dysfunction of the respiratory epithelial barrier. In mouse infection models, intranasal challenge with P. multocida and S. pneumoniae, a well-known respiratory pathogenic bacterium associated with respiratory disorders in humans (26), induced more Evans blue quantified in the tracheae and lungs of bacterium-infected mice than in PBS-treated mice (Fig. 1). As an azo dye that has a very high affinity for serum albumin, Evans blue is used to assess changes in the permeability of physiological barriers such as the blood-brain barrier, intestinal barrier, and respiratory barrier in different disease models (11–13). We also detected increased levels of VEGFA, vWF, and eNOS in the tracheae and lungs of mice challenged with P. multocida compared to those in the main respiratory organs of PBS-treated mice (Fig. 1 and 2). Among these three proteins, increased vWF generally results in increased vascular permeability (27, 28). VEGFA is recognized as a regulatory mediator of vascular permeability, promoting vascular permeability by activating two receptors, VEGFR-1 and VEGFR-2 (29). In many diseases, the increased expression of VEGFA indicates increased vascular permeability (11–13). Since the protein levels of eNOS are upregulated in response to VEGFA, increased expression of eNOS indicates an increase in VEGFA expression (10). Taken together, the above-described findings suggest that P. multocida infection may increase the permeability of the respiratory epithelial barrier.

Cell-cell junctions between epithelial cells, particularly TJs and AJs, are known to play an essential role in maintaining host epithelial permeability (30, 31). Changes in the expression of TJs and AJs between epithelial cell monolayers could also be used to evaluate the conditions of epithelial permeability (32, 33). In this study, our *in vitro* tests revealed that P. multocida infection decreased the expression of several key proteins at TJs (ZO-1) and AJs (β-catenin and E-cadherin) in MLE-12 cells (Fig. 3), confirming that P. multocida infection induces increased respiratory epithelial permeability. Our *in vitro* tests in MLE-12 cells also demonstrated increases in VEGFA and HIF-1α in bacterium-infected cells (Fig. 4), indicating that HIF-1α–VEGFA signaling is activated by P. multocida infection. Notably, when the expression of HIF-1α was suppressed, decreased levels of VEGFA and ZO-1 were observed (Fig. 4). As an important signaling pathway involved in perceiving hypoxia, the HIF-1α–VEGF axis has been proposed as an important mechanism driving epithelial barrier disruption and the increased permeability of the epithelium (34). This finding has been confirmed for many diseases associated with pathogen infection (12, 13). In agreement with the results of those studies, our findings indicate that respiratory infection induces respiratory epithelial permeability by activating HIF-1α–VEGFA signaling.

Since the above-mentioned findings suggest an important role of HIF-1α–VEGFA signaling in the pathogenesis of respiratory bacterial infection, we explored whether HIF-1α or VEGFA could be used as a target to relieve P. multocida infection. Our *in vivo* tests in mouse models showed that treatment using an inhibitor of VEGFR-2 (an important receptor of VEGFA) or an inhibitor of eNOS reduced the severity of infection and tissue damage caused by P. multocida infection, while treatment with an agonist of HIF-1α increased infection and damage (Fig. 5). All of these findings suggest that HIF-1α and/or VEGFA represents a potential target to reduce the severity of infections caused by P. multocida.

While infections caused by P. multocida in humans are well documented (14, 35–37), little is known about the mechanisms involved. Our recent work revealed that P. multocida isolates from different animals demonstrate the capacities to adhere to and invade human respiratory epithelial cells (BEAS-2B) (25). Using BEAS-2B cells as a model, we demonstrated that infections by P. multocida isolates from different animals could induce an increase in respiratory epithelial permeability, and this process was also associated with the bacterial activation of HIF-1α–VEGFA signaling (Fig. 6).

In summary, our results revealed that P. multocida infection induces the activation of HIF-1α–VEGFA signaling that promotes an increase in host respiratory epithelial permeability. Moreover, we demonstrated that P. multocida infection increased the permeability of human respiratory epithelial cells, which was associated with the activation of HIF-1α–VEGFA signaling, suggesting that this may be a mechanism contributing to the pathogenesis of P. multocida infection in humans. We further confirmed that intervention of HIF-1α–VEGFA signaling could mitigate infections caused by respiratory bacteria in mouse models, thereby presenting this pathway as a suitable candidate for therapy against respiratory bacterial infections.

## MATERIALS AND METHODS

### Bacterial strains, cell lines, and culture conditions.

P. multocida strains used in this study were isolated from multiple host species: strain HuN001 (GenBank accession no. CP073238) is a capsular genotype A strain recovered from a patient with pneumonia (38), HN05 (GenBank accession no. PPVF00000000) is a capsular genotype D strain recovered from the trachea of a pig with pneumonia (39), HN06 (GenBank accession no. CP003313) is a toxigenic strain of capsular genotype D recovered from the nasal swabs of a pig with atrophic rhinitis (40), HB01 (GenBank accession no. CP006976) is a capsular type A strain isolated from the lung of a cow with bovine respiratory disease (41), and C48-1 is a capsular genotype A strain recovered from fowl cholera and was a gift from Qingping Luo of the Hubei Academy of Agricultural Sciences. S. pneumoniae D39 was a kind gift from Qi Huang of Huazhong Agricultural University and was used as a quality control strain in this study. Unless otherwise specified, P. multocida and S. pneumoniae strains were cultured on tryptic soy agar (TSA; Becton, Dickinson and Company, MD, USA) or in tryptic soy broth (TSB; Becton, Dickinson and Company, MD, USA) containing 5% bovine serum albumin (BSA) at 37°C for a minimum of 12 h. MLE-12 cells were maintained in a Dulbecco’s modified Eagle’s medium (DMEM)–Ham’s F-12 medium nutrient mixture (Gibco, USA) containing 2% fetal bovine serum (FBS; Bio-Channel, China), 1% GlutaMAX-1, 1 M 1% HEPES buffer solution, 1% penicillin-streptomycin, 1% ITS (insulin-transferrin-selenium), 10 nM hydrocortisone, and 10 nM estradiol at 37°C under a 5% CO_2_ atmosphere, while BEAS-2B cells were maintained in DMEM containing 10% FBS and 1% penicillin-streptomycin at 37°C under a 5% CO_2_ atmosphere.

### Animal tests and ethics statements.

Mouse experiments were conducted at the Laboratory Animal Center of Huazhong Agricultural University (Wuhan, China) with approval from the Institutional Ethics Committees (IECs) of the university (approval no. HZAUMO-2021-0142). Laboratory animals were treated according to regulations on the administration of laboratory animals in Hubei Province (2005). Before the formal experiments, pretests in C57BL/6 mice (~5 to 6 weeks old) were performed to determine the doses of P. multocida strains HuN001 and HN05 as well as S. pneumoniae D39 for challenge. Afterward, each of the experimental mice (C57BL/6; ~5 to 6 weeks old; 5 mice per group) was intranasally challenged with HuN001 (15 CFU), HN05 (5 × 10^7^ CFU), D39 (5 × 10^7^ CFU), or PBS (100 μL). To assess the vascular permeability of the tracheae and lungs in challenged mice, three mice in each group were randomly selected, and each mouse was challenged with Evans blue (30 mg/kg of body weight; Sigma, USA) through the tail vein at 48 h postchallenge (hpc). After 40 min, the mice were euthanized, and dyes in the tracheae and lungs were extracted using formamide (2 mL) at 55°C for 24 h (Fig. 1A). Changes in vascular permeability were evaluated by measuring the absorbance values (optical density at 620 nm [OD_620_]) of the solutions, as described previously (12). In parallel, the remaining two mice without tail vein injection of Evans blue were also euthanized, and their tracheae and lungs were collected for immunohistochemical examinations (Fig. 1A). The above-described experiments were performed three times separately. Final data were presented based on the quantification of Evans blue in the tracheae and lungs of nine mice collected from the three separate tests (Fig. 1C and D).

Two other separate mouse experiments were performed to investigate the effect of blocking or promoting the HIF-1α–VEGFA pathway on respiratory bacterial infections in mice. In the first experiment, 20 mice were intranasally challenged with HN05 (7.5 × 10^8^ CFU per mouse), and 10 additional mice received an intranasal inoculation of PBS (50 μL per mouse). At 4 hpi, 1 dpi, and 2 dpi, each of the 10 mice challenged with HN05 received treatment with SU1498 (30 mg/kg) (inhibitor of VEGFR-2) (catalog no. 168835-82-3; AdooQ BioScience, USA), while the remaining 10 mice challenged with HN05, as well as the 10 PBS-inoculated mice, were treated with PBS (100 μL per mouse) using the same routine (Fig. 5A). The surviving mice in the SU1498-treated and PBS-treated groups were euthanized, and their tracheae and lungs were collected for histological examination. In the second experiment, 30 mice were divided into three groups (10 mice per group), and each mouse was intranasally challenged with HN05 (5 × 10^8^ CFU). At 4 hpi, 1 dpi, and 2 dpi, each mouse in different groups received treatment with DMOG (400 mg/kg) (agonist of HIF-1α) (catalog no. A13998; AdooQ BioScience, USA), L-NAME (20 mg/kg) (inhibitor of eNOS) (catalog no. GA11233; GlpBio, USA), or PBS (100 μL) by intraperitoneal injection (Fig. 5A). Mortalities were recorded every day for 7 days after bacterial infection.

### Histological and immunohistochemical examinations.

Murine tracheae and lungs were fixed using 4% buffered paraformaldehyde for 2 days and routinely processed for embedding in paraffin and sectioning at 3 to 5 μm. For histological examination, tissue sections were dewaxed by step treatments using xylene I (20 min), followed by treatment with xylene II (20 min), xylene III (20 min), anhydrous ethanol I (5 min), anhydrous ethanol II (5 min), 95% alcohol (5 min), 90% alcohol (5 min), 80% alcohol (5 min), and 70% alcohol (5 min). After soaking in distilled water for 5 min, the sections were stained using Harris hematoxylin for 2 min, differentiated with alcohol hydrochloride for 1 s, and washed using water. Thereafter, the sections were stained with a 1% water-soluble eosin solution for 3 min and then washed using water for 30 s. Following dehydration by step treatments using anhydrous ethanol I (5 min), anhydrous ethanol II (5 min), *N*-butanol (5 min), xylene I (5 min), and xylene II (5 min), the sections were air dried and sealed using neutral gum for examination under a microscope. For immunohistochemical examination, dewaxed sections were dehydrated and heated in a microwave oven in citrate buffer for 15 min for antigen retrieval. After blocking in 3% hydrogen peroxide for 15 min, the sections were overlaid with 10% normal goat serum for 30 min. Following this, the sections were incubated with von Willebrand factor (vWF) antibody (1:100) (Abcam, UK) or eNOS polyclonal antibody (1:100) (Invitrogen, USA) at 4°C overnight. The sections were then incubated with horseradish peroxidase (HRP)–goat anti-rabbit IgG (diluted 1:300) for 30 min at 37°C and then incubated with 3,3-*N*-diaminobenzidine tetrahydrochloride (DAB). Finally, the sections were stained with Harris hematoxylin, dehydrated, and sealed for microscopy examination.

### Dextran-based transwell permeability assays.

Dextran-based transwell permeability assays were performed according to protocols documented previously (13, 42). Briefly, approximately 1 × 10^5^ cells (MLE-12 or BEAS-2B) were seeded onto 24-well-plate cell culture inserts (Labselect, Hefei, China) and cultured in antibiotic-free medium (as described above) for 36 h. The cells were incubated with 200 μL of antibiotic-free medium containing bacteria (HuN001, HN05, or D39) at a multiplicity of infection (MOI) of 200 and 70-kDa fluorescein isothiocyanate (FITC; Sigma, St. Louis, MO, USA) (10 μM) at 37°C for 12 h under a 5% CO_2_ atmosphere. Finally, 100 μL of medium from the basal chamber was transferred to a black-well plate (Greiner Bio-One, Germany). Dextran permeability was determined based on the results of reading the plate in a Victor Nivo multimode plate reader (PerkinElmer, Waltham, MA, USA). A 490/520-nm excitation/emission wavelength was utilized for reading fluorescence, as described previously (13).

### Quantitative real-time PCR.

Monolayers of MLE-12 and/or BEAS-2B cells were infected with P. multocida HuN001 or HN05 (MOI of 200) and incubated at 37°C for 6 h. Cells treated with S. pneumoniae D39 (OD_600_ = 0.6) or PBS were used as controls. After that, cells were washed with PBS, and total RNAs were extracted using the TRIzol reagent protocol (Invitrogen, Thermo Fisher, Waltham, MA, USA). cDNAs synthesized from the extracted total RNAs using a PrimeScript RT reagent kit with gDNA Eraser (TaKaRa, Japan) were used as the templates to perform quantitative real-time PCR (qPCR) assays for checking the transcription levels of VEGFA, HIF-1α, ZO-1, β-catenin, E-cadherin, and occludin. Glyceraldehyde-3-phosphate dehydrogenase (GAPDH) was used as a reference gene. Primers used for qPCR are listed in Table S1 in the supplemental material. All experiments were repeated three times.

### Western blotting.

Bacterium-infected monolayers of MLE-12 and/or BEAS-2B cells were lysed using radioimmunoprecipitation assay (RIPA) buffer (Beyotime, China) containing protease inhibitors. The products were then centrifuged at 4°C at 12,000 rpm for 10 min, and the concentrations of proteins in the supernatants were measured using a bicinchoninic acid (BCA) protein assay kit (Beyotime, China). After that, the proteins were separated on a 10% sodium dodecyl sulfate-polyacrylamide gel electrophoresis (SDS-PAGE) gel and then transferred onto a polyvinylidene difluoride (PVDF) membrane (Bio-Rad, USA). The blots were blocked in 5% BSA in Tris-buffered saline with Tween 20 (TBST) for 3 h at room temperature. Following this, the blots were incubated overnight at 4°C with either VEGFA polyclonal antibody (1:2,000) (catalog no. 19003-1-AP; Proteintech, China), ZO-1 polyclonal antibody (1:2,000) (catalog no. 21773-1-AP; Proteintech, China), E-cadherin monoclonal antibody (1:1,000) (catalog no. P12830; Cell Signaling, USA), β-catenin polyclonal antibody (1:5,000) (catalog no. 21773-1-AP; Proteintech, China), HIF-1α antibody (1:200) (catalog no. NB100-134; Novus Biologicals, USA), β-actin monoclonal antibody (1:5,000) (catalog no. 66009-1-lg; Proteintech, China), or GAPDH monoclonal antibody (1:20,000) (catalog no. 60004-1-lg; Proteintech, China). After washing, the blots were incubated with species-specific horseradish peroxidase-conjugated antibodies and visualized with enhanced chemiluminescence (ECL) reagents (Beyotime, China). All Western blots were quantified using ImageJ software, and the results were analyzed as the relative immunoreactivity of each protein normalized to the respective loading controls. Since HIF-1α is not stable and is easily degraded in an aerobic environment (43), samples for the detection of HIF-1α were prepared as follows: cells were incubated in an anaerobic chamber for 6 h after bacterial inoculation, and cell lysis was completed within 10 min.

### siRNA transfection and bacterial infection.

Three specific small interfering RNAs (siRNAs) against HIF-1α (see Table S1 in the supplemental material) were used to suppress the expression of HIF-1α in MLE-12 cells, and according to their efficacy (Fig. 4E), one of the specific siRNAs (forward primer 5′-CCA UGU GAC CAU GAG GAA ATT-3′ and reverse primer 5′-UUU CCU CAU GGU CAC AUG GAT-3′) was selected for suppression. This siRNA (20 nM) was transfected into MLE-12 cells using Lipofectamine 2000 reagent (Invitrogen, USA) according to the manufacturer’s instructions. Scrambled RNA at the same concentration was also transfected as a control. Afterward, monolayers of both siRNA-transfected cells and control cells were infected with P. multocida HuN001 and HN05 (MOI of 200) for 5 h. The cells were lysed using protease inhibitors in RIPA buffer (Beyotime, China). The expression levels of VEGFA and ZO-1 in bacterium-infected cells were determined using Western blotting as described above. This experiment was repeated three times.

### Immunofluorescence.

To observe the expression of HIF-1α in cells during bacterial infection, monolayers of MLE-12 and/or BEAS-2B cells were incubated with P. multocida HuN001 or HN05 (MOI of 200) for 5 h, followed by washing using precooled PBS 3 times to remove the free bacteria. Bacterium-infected cells were then fixed using precooled formaldehyde for 2 h and blocked in 5% BSA for 2 h at room temperature. Thereafter, the cells were incubated with HIF-1α antibody (1:200) overnight at 4°C. After washing, the cells were incubated with Cy3-labeled goat anti-rabbit IgG (catalog no. AS007; ABclonal, USA) at 37°C for 30 min in a dark place. Finally, the cells were incubated with antifade mounting medium containing 4′,6-diamidino-2-phenylindole (DAPI) (Beyotime, China) for 30 min at room temperature in a dark place, and the expression of HIF-1α was observed under an inverted fluorescence microscope.

### Confocal laser scanning microscopy examination.

To observe the expression of ZO-1 in cells during bacterial infection, monolayers of MLE-12 cells on confocal dishes were infected with P. multocida HN05 (MOI of 200) for 5 h, followed by washing using precooled PBS three times to remove the free bacteria. Bacterium-infected cells were then fixed using precooled formaldehyde for 2 h and blocked in 5% BSA for 2 h at room temperature. Thereafter, the cells were incubated with ZO-1 polyclonal antibody (1:2,000) overnight at 4°C. After washing, the cells were incubated with Cy3-labeled goat anti-rabbit IgG at 37°C for 30 min in the dark. Finally, the cells were incubated with antifade mounting medium containing DAPI (Beyotime, China) for 30 min at room temperature in the dark, and the expression of ZO-1 was observed under a superresolution confocal laser scanning microscopy system. The obtained photos were analyzed using NIS-Elements Viewer 4.20 software (Nikon, Tokyo, Japan).

### Statistical analysis.

Statistical analysis was performed using the multiple-*t*-test strategy in GraphPad Prism 8.0 (GraphPad Software, San Diego, CA). Data represent means ± standard deviations (SD). The significance level was set at a *P* value of <0.05 (*), a *P* value of <0.01 (**), or a *P* value of <0.001 (***).
