# Supplementary material for: Vascular Endothelial Growth Factor A Contributes to Increased Mammalian Respiratory Epithelial Permeability Induced by Pasteurella multocida Infection
Source: Microbiol Spectr. 2023 Mar 14;11(2):e04554-22. doi: 10.1128/spectrum.04554-22 (PMC10101004; doi:10.1128/spectrum.04554-22)
Supplement: Supplemental file 1 — Table S1. Download spectrum.04554-22-s0001.docx, DOCX file, 0.02 MB [file spectrum.04554-22-s0001.docx]

**Table S1 Oligonucleotides used in this study.**

| Target genes | Sequences (5’-3’) | Role |
| --- | --- | --- |
| siRNA-1 | F: CCAUGUGACCAUGAGGAAATT | siRNA for suppressing the expression of HIF-1α in MLE-12 cells |
|  | R: UUUCCUCAUGGUCACAUGGAT |  |
| siRNA-2 | F: GCAGACCCAGUUACAGAAATT | siRNA for suppressing the expression of HIF-1α in MLE-12 cells |
|  | R: UUUCUGUAACUGGGUCUGCTG |  |
| siRNA-3 | F: GCAGGAAUUGGAACAUUAUTT | siRNA for suppressing the expression of HIF-1α in MLE-12 cells |
|  | R: AUAAUGUUCCAAUUCCUGCTG |  |
| Murine VEGFA | F: CCGCAGACGTGTAAATGTTCCT | Detecting the transcription of VEGFA in MLE-12 cells |
|  | R: TTCCGGTGAGAGGTCTGGTTC |  |
| Murine HIF-1α | F: GGACAGAGCCGGCGTTTA | Detecting the transcription of HIF-1α in MLE-12 cells |
|  | R: CGGCCCGGCTTACTTTTTCTT |  |
| Murine GAPDH | F: GCACAGTCAAGGCCGAGAAT | Detecting the transcription of GAPDH in MLE-12 cells |
|  | R: GCCTTCTCCATGGTGGTGAA |  |
| Human VEGFA | F: CCCAGTTTTGGGAACACCGA | Detecting the transcription of VEGFA in BEAS-2B cells |
|  | R: CCCCAAAGCACAGCAATGTC |  |
| Human HIF-1α | F: CCATGCCCCAGATTCAGGAT | Detecting the transcription of HIF-1α in BEAS-2B cells |
|  | R: TGGGTTCTTTGCTTCTGTGTCT |  |
| Human GAPDH | F: GGAGTCCACTGGCGTCTTCA | Detecting the transcription of GAPDH in BEAS-2B cells |
|  | R: GTCATGAGTCCTTCCACGATACC |  |
| ZO-1 | F: AAGAGATGAACGGGCTACGC | Detecting the transcription of ZO-1 in BEAS-2B cells |
|  | R: GGAGGCCTATCGTGTGATCG |  |
| β-catenin | F: GCTGGGACCTTGCATAACCT | Detecting the transcription of β-catenin in BEAS-2B cells |
|  | R: CGCACTGCCATTTTAGCTCC |  |
| E-cadherin | F: CTTTGACGCCGAGAGCTACA | Detecting the transcription of E-cadherin in BEAS-2B cells |
|  | R: TTTGAATCGGGTGTCGAGGG |  |
| Occludin | F: GCAAAGTGAATGACAAGCGG | Detecting the transcription of Occludin in BEAS-2B cells |
|  | R: AAGTCATCCACAGGCGAAGT |  |
